# Compliance Challenges in a Longitudinal COVID-19 Cohort Using Wearables for Continuous Monitoring: Observational Study

**DOI:** 10.2196/43134

**Published:** 2023-04-05

**Authors:** Mario Mekhael, Chan Ho, Charbel Noujaim, Ala Assaf, Hadi Younes, Abdel Hadi El Hajjar, Humza A Chaudhry, Brennan Lanier, Nour Chouman, Noor Makan, Botao Shan, Yichi Zhang, Lilas Dagher, Omar Kreidieh, Nassir Marrouche, Eoin Donnellan

**Affiliations:** 1 Tulane University School of Medicine New Orleans, LA United States; 2 Department of Medicine Cleveland Clinic Foundation Cleveland, OH United States; 3 Department of Medicine Emory University Atlanta, GA United States

**Keywords:** COVID-19, digital health, wearables, compliance, cardiovascular health, heart disease, wearable device, biometric, remote monitoring

## Abstract

**Background:**

The WEAICOR (Wearables to Investigate the Long Term Cardiovascular and Behavioral Impacts of COVID-19) study was a prospective observational study that used continuous monitoring to detect and analyze biometrics. Compliance to wearables was a major challenge when conducting the study and was crucial for the results.

**Objective:**

The aim of this study was to evaluate patients’ compliance to wearable wristbands and determinants of compliance in a prospective COVID-19 cohort.

**Methods:**

The Biostrap (Biostrap USA LLC) wearable device was used to monitor participants’ biometric data. Compliance was calculated by dividing the total number of days in which transmissions were sent by the total number of days spent in the WEAICOR study. Univariate correlation analyses were performed, with compliance and days spent in the study as dependent variables and age, BMI, sex, symptom severity, and the number of complications or comorbidities as independent variables. Multivariate linear regression was then performed, with days spent in the study as a dependent variable, to assess the power of different parameters in determining the number of days patients spent in the study.

**Results:**

A total of 122 patients were included in this study. Patients were on average aged 41.32 years, and 46 (38%) were female. Age was found to correlate with compliance (*r*=0.23; *P*=.01). In addition, age (*r*=0.30; *P*=.001), BMI (*r*=0.19; *P*=.03), and the severity of symptoms (*r*=0.19; *P*=.03) were found to correlate with days spent in the WEAICOR study. Per our multivariate analysis, in which days spent in the study was a dependent variable, only increased age was a significant determinant of compliance with wearables (adjusted *R*^2^=0.1; *β*=1.6; *P*=.01).

**Conclusions:**

Compliance is a major obstacle in remote monitoring studies, and the reasons for a lack of compliance are multifactorial. Patient factors such as age, in addition to environmental factors, can affect compliance to wearables.

## Introduction

The rapid, ongoing integration of digital technologies in medicine has improved patient management, providing safe, effective, and standardized interventions. Digital tools offer several solutions and benefits that were previously unavailable, promoting beneficial behaviors such as smoking cessation [1,2], physical activity [3], and regulated alcohol consumption [4]. By using wearable technologies, physicians can remotely monitor patients’ vital signs and diagnose several different pathologies. These interventions have improved outcomes in patients with chronic diseases, such as cardiovascular comorbidities [5]. Moreover, wearables have gained popularity, particularly in the field of cardiac electrophysiology. Many large studies have addressed the performance of wearables in detecting arrhythmias, such as atrial fibrillation, and the potential implementation of these digital tools in medical interventions [6,7]. However, other studies have shown that the benefits of digital solutions are limited [8-12]. These findings are primarily the result of decreased compliance or difficulties in using such tools [8,13-15].

Although digital tools are currently facilitating medical practice, many new hurdles have emerged during this process. One of these challenges is patient compliance and adherence to these devices. Compliance is commonly viewed as a standard measurement of the quality of digital health studies. Regardless of the efficacy and performance level of a digital intervention, the latter loses its value in the absence of compliance. Unfortunately, there is still no established universal standard for assessing compliance. For example, Christensen et al [16] defined *adherence* as the extent of an individual’s experience with the intervention. However, this definition does not consider the World Health Organization’s initial conclusions that adherence is the extent to which a patient follows medical instructions. They elaborated that the term *medical* limits the extent of interventions that patients with long-lasting diseases receive. Additionally, the term *instructions* implies passivity on the patient side and might imply that patients are only recipients rather than collaborators [17]. Therefore, to establish adherence, several edits to the definition were made to accurately describe the concept and incorporate both patients’ experiences and physicians’ goals [13].

The WEAICOR (Wearables to Investigate the Long Term Cardiovascular and Behavioral Impacts of COVID-19) study was a prospective observational study that used continuous monitoring to detect and analyze biometrics [18]. While performing this study, several challenges related to adherence to wearables were faced, and several techniques to improve compliance were used.

The aim of this study was to evaluate patients’ compliance and the determinants of this compliance in a prospective COVID-19 cohort.

## Methods

### Evaluation of Compliance in a Prospective COVID-19 Cohort

The WEAICOR study was a prospective observational study of patients who were aged 18 years or older and were monitored via the Biostrap (Biostrap USA LLC) wearable wristband device. The study aimed to identify the impact of the post–COVID-19 condition on sleep by using wearables. In this analysis, we sought to evaluate patient compliance.

After eligibility screening and the signing of electronic consent forms, all patients were sent a Biostrap device by mail to continuously monitor their biometric data, including heart rate, heart rate variability, respiratory rate, and oxygen saturation. Instructions on how to use the device were provided via phone calls by the study coordinator, along with a recorded video that detailed the steps for activating the device and linking it to the mobile app. Participants were instructed to wear the smart band.

### Ethics Approval

This study was approved by Tulane University Institution Review Board on June 9, 2020 (study #2020-678).

### Study Population

In this analysis, patients who recovered from COVID-19 were included. Participants who had a positive COVID-19 diagnosis were recruited via flyers and advertisements on different social media platforms and via mass emails that were generated and sent to Tulane University’s staff and student body. A total of 200 patients were assessed for eligibility by September 2021. Participants received a baseline questionnaire that collected demographic and medical history data. We secured informed consent forms and listed Tulane University as an organization with data access.

### Biostrap Device

The Biostrap is a photoplethysmography-based smart band that records patients’ vitals at rest and at 5-minute intervals and generates graphic results and reports on the Biostrap mobile app. The device has a life span of 2.5 days and is splashproof. Patients were able to keep the device after the completion of the WEAICOR study. The mobile app transferred photoplethysmography and motion signal data collected from the wrist to the Biostrap cloud server, where the data underwent signal processing and analysis via machine learning algorithms to generate resting physiological data, which were transferred to Tulane University’s data server. The data were automatically synchronized in real time and could be viewed on the Biostrap portal by the research team. The study participants used their own smartphones. The accuracy and reproducibility of the Biostrap device in assessing basic physiological data were already reported in previously published studies [19,20]. Moreover, those studies and ours relied on the raw photoplethysmography signals obtained from the same type of device to extract parameters of interest; thus, the accuracy and reproducibility reported in those studies are applicable to the findings of our study.

### Patient Follow-up

The Biostrap remote monitoring system ensured data generation, compliance, and technical troubleshooting throughout the WEAICOR study. The research coordinator explained to the participants the purpose of the study, the risks accompanied with the study, the ability to drop out at any time during the study, and the types of data that were being collected (eg, raw photoplethysmography signals, heart rate, heart rate variability, oxygen saturation, respiratory rate, etc). Patients were called regularly by the study coordinator to assess for problems or compliance issues. The compliance of participants was also assessed throughout the study. Noncompliant patients were either contacted by the research coordinator or prompted via cell phone notifications to wear the device. Data on symptoms were collected by using surveys, which were collected at different time points (2 weeks, 1 month, 6 months, and 12 months).

### Data Analysis

A wearable device was used to monitor participants’ biometric data. Analyses were performed by using prospectively obtained continuous wearable data. Adherence to the wearable device was based on the following two metrics: compliance and days spent in the WEAICOR study. Compliance was calculated as a percentage by dividing the total number of days in which data were transmitted by the total number of days spent in the study. Because compliance could take any data value between 0 and 100, it was considered a continuous variable for the analysis. We also calculated the total number of days patients remained in the study.

A univariate correlation analysis was performed, with compliance as a dependent variable and age, BMI, sex, symptom severity, and the number of complications or comorbidities as independent variables. Symptom severity was calculated on a scale of 1 to 5, with 1 indicating no symptoms or minor symptoms and 5 indicating very severe symptoms. The number of complications was the sum of the number of comorbidities in each patient. For example, for a patient with hypertension and diabetes who has no other risk factors, the number of complications or comorbidities would be 2.

The same analysis was performed with days spent in the study as a dependent variable. Since multiple independent variables correlated with the days spent in the study, backward stepwise multivariate linear regression was performed, with days spent in the study as a dependent variable, to assess the power of different parameters in determining the number of days patients spent in the study. No multivariate analysis was performed with compliance as a dependent variable, as only age was significant in the univariate analysis (*r*=0.23; *P*=.01).

## Results

### Baseline Characteristics and Compliance

**Figure 1:**
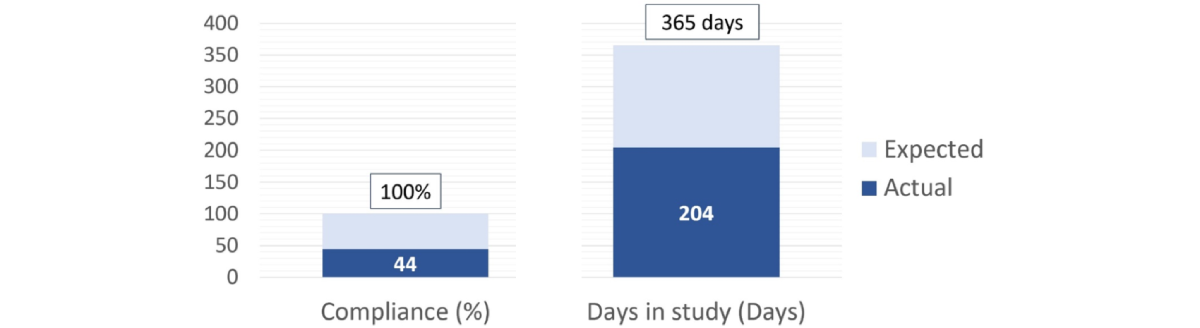
Average compliance (%) and days spent in the study for the study population.

**Figure 2:**
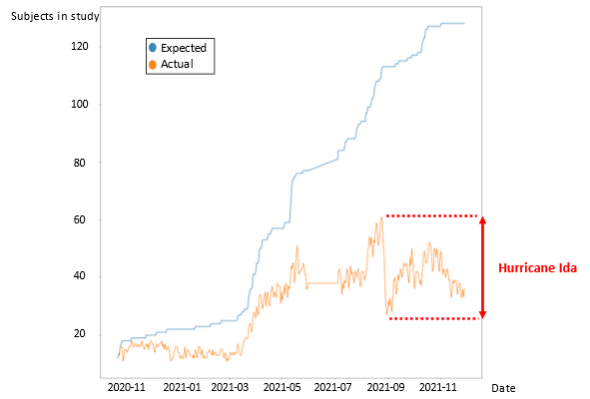
Expected and actual compliance evolution during the study. The difference between expected and actual compliance represents noncompliant patients.

A total of 122 patients were included. Patients were on average aged 41.32 (SD 15.7) years, and 46 (38%) were female. The population tended to be young and had few or no comorbidities. Most of the participants (112, 92%) were not hospitalized during their COVID-19 course. Baseline demographics are detailed in Table 1. On average, patients had a compliance rate of 44.62% (SD 33.54%) and were followed up for 203.78 (SD 98.58) days (Figure 1). Compliance decreased with the progression of the WEAICOR study, with a marked acute drop in compliance on the day that Hurricane Ida occurred in New Orleans (Figure 2). The research coordinator that followed up with the patients noted no problems regarding skin reactions or allergies to the device and no server or device manufacturer issues.

**Table 1:** Baseline demographic and clinical characteristics of the COVID-19 arm (N=122).

Characteristics			COVID-19 group
Age (years), mean (SD)			41.32 (15.7)
**Sex, n (%)**			
	Male	76 (62)	
	Female	46 (38)	
BMI (kg/m^2^), mean (SD)			28.7 (8.6)
**Race, n (%)**			
	Caucasian or White	71 (58)	
	African American or Black	20 (16.5)	
	Asian	12 (10)	
	Latino or Hispanic	5 (4.5)	
	Other	13 (11)	
**Comorbidities, n (%)**			
	None	88 (72)	
	Diabetes	6 (5)	
	Immune system deficiencies or HIV	1 (1)	
	Heart conditions	4 (3)	
	Asthma or chronic lung disease	15 (12)	
	Extreme obesity	5 (4)	
	Cancer treatment	4 (3)	
**Education level, n (%)**			
	Bachelor’s degree	27 (22)	
	Some college	30 (24)	
	Associate degree	16 (13)	
	Master’s degree	28 (23)	
	Doctorate	1 (1)	
	Professional	10 (8)	
	Other	11 (9)	

### Univariate Analysis

**Figure 3:**
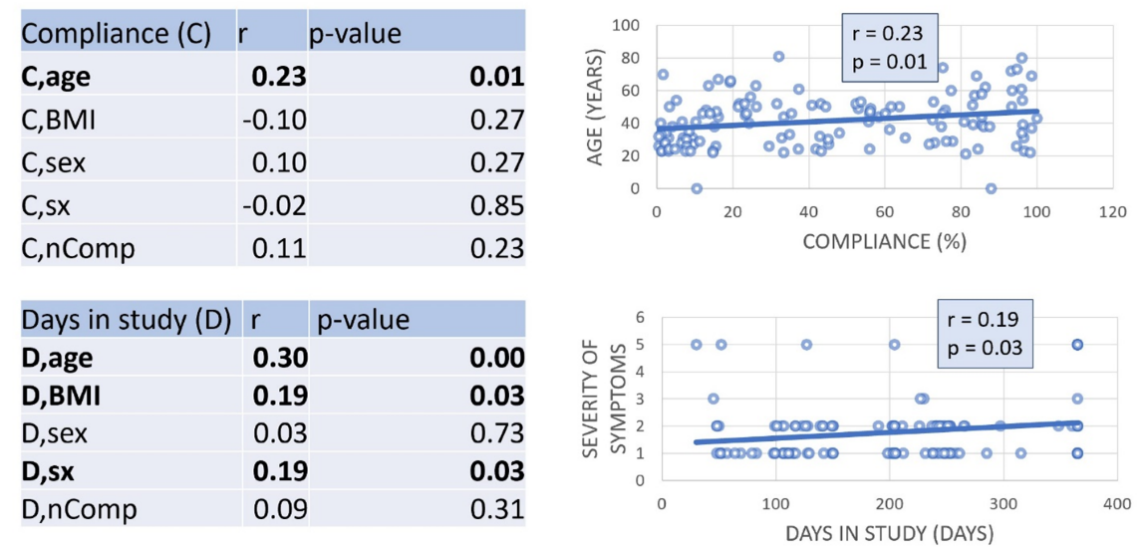
Univariate regressions with compliance and days in the study as dependent variables and age, BMI, sex, sx, and nComp as independent variables. nComp: number of comorbidities; sx: severity of symptoms.

Only age was found to correlate with compliance (*r*=0.23; *P*=.01). The number of complications (*r*=0.11; *P*=.23), BMI (*r*=−0.10; *P*=.27), sex (*r*=0.10; *P*=.27), and the severity of symptoms (*r*=−0.02; *P*=.85) were not found to be determinants of compliance. In addition, age (*r*=0.30; *P*=.001), BMI (*r*=0.19; *P*=.03), and the severity of symptoms (*r*=0.19; *P*=.03) were found to correlate with days spent in the WEAICOR study (Figure 3). The number of complications (*r*=0.09; *P*=.31) and sex (*r*=0.03; *P*=.73) were not found to be determinants of increased days spent in the study (Figure 3).

### Multivariate Analysis

Per our backward stepwise multivariate analysis, in which days spent in the WEAICOR study was a dependent variable, only increased age was a significant determinant of increased days spent in the study (adjusted *R*^2^=0.1; *β*=1.6; *P*=.01). BMI (*P*=.07), the severity of symptoms (*P*=.25), complication count (*P*=.47), and sex (*P*=.86) were nonsignificant determinants of increased days spent in the study (Table 2).

**Table 2:** Multivariate linear regression (adjusted *R*^2^=0.1) with days spent in the study as a dependent variable and age, BMI, sex, the severity of symptoms, and the number of comorbidities as independent variables.

Variables	Coefficients, *β* (SE; 95% CI)	*P* value
Age	1.61 (0.58; 0.46 to 2.76)	.01
Sex	3.07 (17.81; −32.20 to 38.33)	.86
BMI	1.92 (1.05; −0.17 to 4.01)	.07
Severity of symptoms	9.38 (8.19; −6.82 to 25.59)	.25
Complication count	−6.10 (8.44; −22.81 to 10.62)	.47

## Discussion

### Principal Findings

In our study, we report several findings. First, advanced age was the most significant factor in increasing compliance with wearables (*r*=0.23; *P*=.01). However, the power of the association was weak, suggesting unknown determinants. Second, patients with increased symptom severity were more likely to be compliant to a wearable device. Third, environmental factors, such as natural disasters, can affect wearable compliance during remote studies.

### Defining the Concept of Compliance

Digital remote management is accompanied by hurdles that must be addressed. One of the significant challenges that was faced during remote management in the WEAICOR study was patients’ compliance with the device. In clinical management, decreased compliance with a digital device can disturb therapeutic strategies and result in physicians missing clinical events. Additionally, from a research perspective, reduced adherence can adversely impact results [21]. For this reason, it is recommended that authors report levels of nonadherence when citing results, so that readers can have an accurate estimate of performance [22].

The concept of compliance is not very well defined. Many discrepancies exist in the ways that physicians and researchers describe adherence to a digital device. Many authors believe that compliance should be measured and scaled based on patients’ experiences [16]. Others believe that compliance should be viewed from a physician’s perspective and based on objective clinical assessments and scales to have reliable quantitative measures. Recently, the importance of both perspectives has started to be appreciated, and definitions combining both viewpoints have emerged [13]. In our study, compliance was defined quantitatively, using objective transmitted data. This allowed us to generate a reliable measurement tool independent of patients’ and physicians’ perspectives. However, the assessment and fine-tuning of this objective tool should be constantly subjected to feedback from both patients and physicians. Future studies should focus on standardizing compliance measurement while also taking into account patients’ and physicians’ feedback to maximize adherence.

### Determinants of Compliance With Wearables

The severity of symptoms (*r*=0.19; *P*=.03), age (*r*=0.30; *P*=.001), and BMI (*r*=0.19; *P*=.03) were significant in determining the number of days patients spent in our study. Additionally, as seen in the multivariate analysis, age was the strongest predictor. The influences of age and patient-related factors on compliance rates and days spent in the study emphasize the fact that compliance to a wearable is multifactorial and cannot be well defined.

Studies are conflicting with regard to the relationship between age and compliance. Although some studies have shown that medication adherence increases with age [23,24], others have demonstrated reduced adherence in older patients [25]. Kim et al [26] described an inverted *U*-shaped pattern for age and compliance, with maximum compliance in those aged 60 to 69 years and decreased compliance for the extremes of age. Our population’s mean age was younger; therefore, the trend of compliance increasing with age is in agreement with previous studies. A subanalysis of the TeleCheck-AF study showed that compliance increased with age [27]. In general, this tendency can be explained by several factors. For example, older patients tend to be more careful in matters related to their health, reflecting increased interaction with the health care system [24], but this improved compliance can be limited by several parameters, specifically those in the older population. For instance, older patients might have problems with accessing digital health information and using digital devices [28]. However, in our study, patients were only required to wear a wristband, and no major interactions with sophisticated digital tools were required, which might explain the absence of such problems. Further, in a review of the determinants of compliance, more papers showing a positive correlation between compliance and age were identified [29,30].

Patients with more severe symptoms spent more days in the WEAICOR study when compared to patients with lower symptom severity. Previous studies are conflicting with regard to this finding. Similar to our study, Wild et al [31] reported greater compliance among those with increased disease severity. Moreover, in an experiment to learn about how the experiences of patients with conditions of varying severity influenced their selection of hospitals, patients with health conditions of the highest severity appeared to want the best hospital and the best specialist physician to tend to their conditions [32]. This can be explained by the fact that patients with greater disease severity have more insight into their problems and take specific steps to address them. Other studies have shown no difference in compliance [33] or decreased compliance as symptom severity increases [34]. In fact, Matthews and Hingson [33] showed that several factors, such as patient-physician interaction and certain health-related beliefs, might play a more significant role in relation to compliance. In our study, symptom severity was not a significant predictor of compliance in the multivariate analysis (*P*=.25), which may suggest that age might be a potential confounder. These discrepancies support the multifactorial aspects of patient compliance and the need for detailed prospective studies that specifically aim to understand determinants of compliance. Moreover, the low power of association in the multivariate analysis indicates that there remain many unknown determinants of compliance.

Finally, the impact of Hurricane Ida (which occurred in New Orleans) on the WEAICOR study proves that while compliance is intimately related to patient factors, environmental factors can also dramatically affect an entire group's compliance. Since all of the study participants were recruited from Louisiana, from Hurricane Ida’s landfall on August 29, 2021, onward, compliance dropped drastically. The drop in patient compliance after this date is an example of the difficulties associated with continuing routine care after a natural disaster and shows that remote monitoring can be as problematic as medication maintenance in postdisaster health care [35]. This decrease in compliance might have been due to several factors, such as the preoccupation with basic needs when the hurricane occurred (eg, safety, food, etc). Additionally, the lack of electricity following the hurricane and the inability to charge the Biostrap devices may have also affected patients’ adherence. Despite the unexpected effects of a natural disaster, compliance could be improved by sending automated app, text, and call-based reminders to patients and using a patient portal to facilitate communication between patients and physicians [36]. Compliance improvement resulting from reminders can have similar effects independent of the technique used [37].

### Limitations

Our study presents several limitations. First, the recruitment of Tulane University staff and students likely resulted in a highly health literate, younger study population. Our results should be replicated in larger studies that include older patients and patients with a health literacy level that is reflective of the broader population. Second, our study population was mostly comprised of healthy individuals (88/122, 72%). As such, the impact of comorbidities on compliance might not have been well assessed. Third, female patients were underrepresented in this study, which could limit the generalizability of our results. Fourth, even though all participants were technology literate, they were not asked about their previous wearable usage. Finally, subjective and participant-specific factors that may affect participant compliance were not researched and should be the subject of further studies.

### Conclusion

Device compliance is a major obstacle in remote monitoring studies. Patient and environmental factors, including age, symptom severity, and natural disasters, play a role in adherence to wearable devices and can impact study results. Future remote monitoring studies should focus on increasing compliance to achieve more accurate and reliable results.

## Abbreviations

WEAICOR: Wearables to Investigate the Long Term Cardiovascular and Behavioral Impacts of COVID-19

## Data Availability

The data supporting this study's findings are available from the corresponding author, NM, upon reasonable request.

## References

[1] Free C, Knight R, Robertson S, Whittaker R, Edwards P, Zhou W, Rodgers A, Cairns J, Kenward MG, Roberts I (2011). Smoking cessation support delivered via mobile phone text messaging (txt2stop): a single-blind, randomised trial. Lancet.

[2] Zhuo X, Zhang P, Barker L, Albright A, Thompson TJ, Gregg E (2014). The lifetime cost of diabetes and its implications for diabetes prevention. Diabetes Care.

[3] Peyrot M, Rubin RR (2008). Access to diabetes self-management education. Diabetes Educ.

[4] Khadjesari Z, Murray E, Hewitt C, Hartley S, Godfrey C (2011). Can stand-alone computer-based interventions reduce alcohol consumption? A systematic review. Addiction.

[5] Mizuno A, Changolkar S, Patel MS (2021). Wearable devices to monitor and reduce the risk of cardiovascular disease: Evidence and opportunities. Annu Rev Med.

[6] Seshadri DR, Bittel B, Browsky D, Houghtaling P, Drummond CK, Desai MY, Gillinov AM (2020). Accuracy of Apple Watch for detection of atrial fibrillation. Circulation.

[7] Wasserlauf J, You C, Patel R, Valys A, Albert D, Passman R (2019). Smartwatch performance for the detection and quantification of atrial fibrillation. Circ Arrhythm Electrophysiol.

[8] Kelders SM, Kok RN, Ossebaard HC, Van Gemert-Pijnen JEWC (2012). Persuasive system design does matter: a systematic review of adherence to web-based interventions. J Med Internet Res.

[9] Elbert NJ, van Os-Medendorp H, van Renselaar W, Ekeland AG, Hakkaart-van Roijen L, Raat H, Nijsten TEC, Pasmans SGMA (2014). Effectiveness and cost-effectiveness of ehealth interventions in somatic diseases: a systematic review of systematic reviews and meta-analyses. J Med Internet Res.

[10] Beishuizen CRL, Stephan BCM, van Gool WA, Brayne C, Peters RJG, Andrieu S, Kivipelto M, Soininen H, Busschers WB, van Charante EPM, Richard E (2016). Web-based interventions targeting cardiovascular risk factors in middle-aged and older people: A systematic review and meta-analysis. J Med Internet Res.

[11] Hadjiconstantinou M, Byrne J, Bodicoat DH, Robertson N, Eborall H, Khunti K, Davies MJ (2016). Do web-based interventions improve well-being in type 2 diabetes? A systematic review and meta-analysis. J Med Internet Res.

[12] Kooij L, Groen WG, van Harten WH (2017). The effectiveness of information technology-supported shared care for patients with chronic disease: A systematic review. J Med Internet Res.

[13] Donkin L, Christensen H, Naismith SL, Neal B, Hickie IB, Glozier N (2011). A systematic review of the impact of adherence on the effectiveness of e-therapies. J Med Internet Res.

[14] Manwaring JL, Bryson SW, Goldschmidt AB, Winzelberg AJ, Luce KH, Cunning D, Wilfley DE, Taylor CB (2008). Do adherence variables predict outcome in an online program for the prevention of eating disorders?. J Consult Clin Psychol.

[15] Michie S, Yardley L, West R, Patrick K, Greaves F (2017). Developing and evaluating digital interventions to promote behavior change in health and health care: Recommendations resulting from an international workshop. J Med Internet Res.

[16] Christensen H, Griffiths KM, Farrer L (2009). Adherence in internet interventions for anxiety and depression. J Med Internet Res.

[17] World Health Organization (2003). Adherence to Long-term Therapies: Evidence for Action.

[18] Mekhael M, Lim CH, El Hajjar AH, Noujaim C, Pottle C, Makan N, Dagher L, Zhang Y, Chouman N, Li DL, Ayoub T, Marrouche N (2022). Studying the effect of long COVID-19 infection on sleep quality using wearable health devices: Observational study. J Med Internet Res.

[19] Selder JL, Proesmans T, Breukel L, Dur O, Gielen W, van Rossum AC, Allaart CP (2020). Assessment of a standalone photoplethysmography (PPG) algorithm for detection of atrial fibrillation on wristband-derived data. Comput Methods Programs Biomed.

[20] Gielen W, Longoria KA, van Mourik RA (2021). Two cases of COVID-19 monitored by a wearable biosensor-a case report. Mhealth.

[21] Olgin JE, Lee BK, Vittinghoff E, Morin DP, Zweibel S, Rashba E, Chung EH, Borggrefe M, Hulley S, Lin F, Hue TF, Pletcher MJ (2020). Impact of wearable cardioverter-defibrillator compliance on outcomes in the VEST trial: As-treated and per-protocol analyses. J Cardiovasc Electrophysiol.

[22] Eysenbach G (2005). The law of attrition. J Med Internet Res.

[23] Rolnick SJ, Pawloski PA, Hedblom BD, Asche SE, Bruzek RJ (2013). Patient characteristics associated with medication adherence. Clin Med Res.

[24] Cohen MJ, Shaykevich S, Cawthon C, Kripalani S, Paasche-Orlow MK, Schnipper JL (2012). Predictors of medication adherence postdischarge: the impact of patient age, insurance status, and prior adherence. J Hosp Med.

[25] Spaans EAJM, Kleefstra N, Groenier KH, Bilo HJG, Brand PLP (2020). Adherence to insulin pump treatment declines with increasing age in adolescents with type 1 diabetes mellitus. Acta Paediatr.

[26] Kim SJ, Kwon OD, Han EB, Lee CM, Oh SW, Joh HK, Oh B, Kwon H, Cho B, Choi HC (2019). Impact of number of medications and age on adherence to antihypertensive medications: A nationwide population-based study. Medicine (Baltimore).

[27] Gawałko M, Hermans AN, van der Velden RM, Betz K, Verhaert DV, Hillmann HA, Scherr D, Meier J, Sultan A, Steven D, Terentieva E, Pisters R, Hemels M, Voorhout L, Lodziński P, Krzowski B, Gupta D, Kozhuharov N, Pison L, Gruwez H, Desteghe L, Heidbuchel H, Evens S, Svennberg E, de Potter T, Vernooy K, Pluymaekers NA, Manninger M, Duncker D, Sohaib A, Linz D, Hendriks JM (2022). Patient motivation and adherence to an on-demand app-based heart rate and rhythm monitoring for atrial fibrillation management: data from the TeleCheck-AF project. Eur J Cardiovasc Nurs. Epub ahead of print.

[28] Tappen RM, Cooley ME, Luckmann R, Panday S (2022). Digital health information disparities in older adults: a mixed methods study. J Racial Ethn Health Disparities.

[29] Jin J, Sklar GE, Oh VMS, Li SC (2008). Factors affecting therapeutic compliance: A review from the patient's perspective. Ther Clin Risk Manag.

[30] Chouman N, Mekhael M, Noujaim C, Lim CH, El Hajjar AH, Ayoub T, Li DL, Marrouche NF (2022). Predictors of patient compliance to digital ECG device: DECAFF II sub-analysis. Cardiovasc Digit Health J.

[31] Wild MR, Engleman HM, Douglas NJ, Espie CA (2004). Can psychological factors help us to determine adherence to CPAP? A prospective study. Eur Respir J.

[32] Halkes R (2006). De Zorgconsument in opkomst - Een onderzoek naar keuzeprocessen in de gezondheidszorg. Academia.

[33] Matthews D, Hingson R (1977). Improving patient compliance: a guide for physicians. Med Clin North Am.

[34] Kyngäs HA (1999). Compliance of adolescents with asthma. Nurs Health Sci.

[35] Ochi S, Hodgson S, Landeg O, Mayner L, Murray V (2014). Disaster-driven evacuation and medication loss: a systematic literature review. PLoS Curr.

[36] Cohen S, Waks Z, Elm JJ, Gordon MF, Grachev ID, Navon-Perry L, Fine S, Grossman I, Papapetropoulos S, Savola JM (2018). Characterizing patient compliance over six months in remote digital trials of Parkinson's and Huntington disease. BMC Med Inform Decis Mak.

[37] Clarke G, Eubanks D, Reid E, Kelleher C, O'Connor E, DeBar LL, Lynch F, Nunley S, Gullion C (2005). Overcoming Depression on the Internet (ODIN) (2): a randomized trial of a self-help depression skills program with reminders. J Med Internet Res.

